# Tobacco Cessation Treatment for Alcohol-Dependent Smokers: When Is the Best Time?

**Published:** 2006

**Authors:** Molly Kodl, Steven S. Fu, Anne M. Joseph

**Affiliations:** Molly Kodl, Ph.D., is a fellow; Steven S. Fu, M.D., M.S.C.E., is an assistant professor of Medicine; and Anne M. Joseph, M.D., M.P.H., is a professor of Medicine, all at The Center for Chronic Disease Outcomes Research at the Minneapolis VA Medical Center and the University of Minnesota, Minneapolis, Minnesota.

**Keywords:** Alcohol and tobacco, alcohol and other drug (AOD) use, abuse, and dependence, cigarette smoking, nicotine dependence, smoking cessation treatment, treatment outcomes, concurrent treatment, co-treatment, intervention, cessation of AOD use (AODU)

## Abstract

Cigarette smoking is highly prevalent among people with alcohol use disorders. Although several studies have demonstrated the feasibility of treating nicotine dependence in people with substance use disorders, researchers and clinicians continue to debate whether nicotine dependence treatment should be delivered simultaneously with or subsequent to alcohol treatment. Evidence suggests that alcohol-dependent individuals prefer sequential treatment and that simultaneous treatment can negatively impact alcohol use outcomes, although the literature is not conclusive. This review includes recommendations of considerations for treatment timing decisions and future research directions.

Cigarette smoking is highly prevalent among people with alcohol abuse and dependence (i.e., alcohol use disorders) (Hughes 1996). Rates of current smoking range from 35 to 44 percent in population-based studies of adults with alcohol use disorders (Grant et al. 2004; Lasser et al. 2000) and may reach 80 percent in treatment-seeking populations (Hughes 1995). In addition, current alcohol use problems are associated with higher levels of nicotine dependence and a lower likelihood of smoking cessation (Breslau et al. 1996; Hays et al. 1999). Given the increased tobacco-related mortality and morbidity in alcohol-dependent smokers (Hurt et al. 1996) and the enhanced difficulty quitting smoking, identifying the most effective treatments and the optimal timing for treatment is critical.

Researchers and clinicians continue to debate the advisability of simultaneous nicotine and alcohol dependence treatment compared with postponing smoking treatment (Hurt and Patten 2003; Sussman 2002). Theoretical arguments against simultaneous treatment include the possibility that concurrent intervention could be detrimental to alcohol treatment outcomes (Bowman and Walsh 2003; Joseph et al. 2004*b*; Kalman 1998). Another frequently cited reason to avoid concurrent treatment is the belief that alcohol users do not want to quit smoking (Kalman 1998). This article reviews the evidence regarding the effects of smoking cessation treatment on alcohol treatment outcomes and the merit of simultaneous tobacco treatment versus sequential treatment for alcohol-dependent patients. A detailed review of individual studies is beyond the scope of this article, and we refer readers to Prochaska and colleagues (2004), Hughes and Kalman (2006), Hurt and Patten (2003), Kalman (1998), and Sussman (2002) for comprehensive summaries.

## Substance-Dependent Smokers’ Perspectives on Smoking Cessation

Early studies suggested that a minority of people in treatment for alcohol or other drug abuse were interested in smoking cessation (Kozlowski et al. 1989; Monti et al. 1995; Orleans and Hutchinson 1993). As national interest in smoking cessation has grown, however, more smokers in substance abuse treatment are considering quitting smoking (Ellingstad et al. 1999; Rohsenow et al. 2005). Several studies have found that alcohol-dependent smokers express a preference for sequential, rather than simultaneous, tobacco treatment (Ellingstad et al. 1999; Kozlowski et al. 1989; Monti et al. 1995; Orleans and Hutchinson 1993; Rohsenow et al. 2005). This may be especially true for alcohol-dependent smokers who rely on smoking to help them to cope with urges to drink (Sussman 2002).

Other studies suggest that alcohol-dependent smokers are not opposed to concurrent smoking cessation treatment. Asher and colleagues (2003) found that fewer than half of alcohol-dependent smokers believed that quitting smoking would make it harder to maintain sobriety, and only 13 percent believed that if they quit smoking, they would be unable to manage urges to drink or use drugs. Despite this, lack of opposition to simultaneous treatment may not be synonymous with willingness to participate in concurrent smoking cessation treatment (Campbell et al. 1998). Overall, individual preferences regarding treatment timing may depend on the degree of relatedness of smoking and drinking behaviors (Ellingstad et al. 1999; Rohsenow et al. 2005; Sobell et al. 1995). For example, Ellingstad and colleagues (1999) found that patients interested in concurrent treatment, compared with those who preferred to address alcohol dependence first, were more likely to believe that quitting cigarettes would help them to resolve their drinking.

## Treatment Outcomes for Alcohol-Dependent Smokers

There are two important issues to consider regarding smoking intervention for alcohol-dependent smokers: effects on smoking behavior (i.e., abstinence from tobacco) and effects on alcohol treatment outcomes (i.e., abstinence or reduction of alcohol use). This section will review studies of tobacco cessation and substance abuse treatment among people with concurrent smoking and substance abuse, first reviewing studies of concurrent treatment, followed by studies of sequential treatment. For tobacco cessation outcomes among people with prior alcohol use, see the Textbox.

## Studies of Concurrent Treatment

Studies on the effectiveness of treatment for current alcohol use problems and nicotine dependence demonstrate variable results. Bobo and colleagues (1996, 1998) conducted two randomized^1^ trials of smoking intervention among alcoholic smokers. Results showed that receiving a smoking intervention did not have a significant detrimental effect on alcohol use (Bobo et al. 1996, 1998). However, neither study demonstrated significant intervention effects on smoking. Rates of smoking abstinence ranged from 3 to 9 percent for intervention groups and 6 to 7 percent for the control groups at 6- and 12-month follow-up visits (Bobo et al. 1996, 1998). In a nonrandomized study of 101 alcoholics receiving inpatient treatment, those who received a 10-hour group smoking intervention had higher rates of smoking cessation than those in the control group (11.8 percent versus 0.0 percent) and similar alcohol and drug use outcomes 1 year after treatment discharge (Hurt et al. 1994). Finally, Cornelius and colleagues (1999) evaluated the antidepressant fluoxetine versus placebo in the treatment of 42 depressed, alcohol-dependent smokers in a randomized controlled trial. Average cigarette and alcohol consumption both were reduced for those in the treatment group compared with the control group (Cornelius et al. 1997, 1999).

**Tobacco Cessation Outcomes Among People With Prior Alcohol Use**

Early studies (Bobo et al. 1987; Covey et al. 1993), more recent examinations (e.g., Hughes and Callas 2003), and an upcoming comprehensive review (Hughes and Kalman 2006) do not provide evidence that a history of alcohol abuse impacts smoking cessation outcomes. Smokers with past alcohol use disorders appear to be as able to stop smoking as smokers in the general population (Hughes and Kalman 2006).

In contrast, other studies have demonstrated worse substance use outcomes following smoking intervention. For example, Joseph and colleagues (1993) compared smokers at a residential treatment program (68 percent had alcohol as the first drug of choice) who enrolled before or after implementation of a hospitalwide smoking ban and mandatory smoking cessation intervention. At follow-up, averaging 11 to 16 months after treatment, 3 percent of those who enrolled prior to the ban and 10 percent of those enrolled after the ban and who received the smoking intervention were abstinent from cigarettes (the difference was not significant). Results suggested that people who received the smoking intervention had worse substance use outcomes than those who did not^2^ (Joseph et al. 1993).

Grant and colleagues (2003) studied 40 alcohol-dependent veterans in outpatient treatment, half of whom received a 5-week group smoking intervention. Although there were no significant differences in the prevalence of smoking abstinence in the past 7 days at 12 months (100 percent of the intervention group and 93 percent of control subjects were smoking), results suggested increased drinking in the intervention group (e.g., at 1 month, 33 percent of the intervention group and 0 percent of the control group reported having more than one drink) (Grant et al. 2003).

Finally, a recent meta-analysis of 19 randomized controlled trials of smoking cessation intervention for people in treatment for or recovery from an addiction^3^ concluded that there was no detrimental effect on substance use outcomes from combined treatment (Prochaska et al. 2004). Smoking cessation interventions were not successful in achieving long-term smoking abstinence, however, in comparison to the control groups (Prochaska et al. 2004). Prochaska and colleagues’ (2004) review suggests, based on studies of individuals in treatment for or recovering from substance dependence, that treatment for both nicotine and other substance dependence is not harmful. In fact, although the data regarding the impact of continued smoking on maintenance of alcohol abstinence are mixed (Sobell et al. 2002), the findings of the Prochaska and colleagues’ (2004) review suggest that receiving a smoking cessation intervention while in treatment may help with long-term abstinence from alcohol and other drugs.

### Studies of Concurrent Versus Delayed Treatment

Only two known randomized controlled studies have specifically evaluated the effects of providing tobacco cessation treatment concurrently with alcohol dependence treatment versus sequential treatment for tobacco and alcohol (Kalman et al. 2001; Joseph et al. 2004*b*). The Timing of Alcohol and Smoking Cessation (TASC) study, which had 499 participants, 68 percent of whom were male, was a clinical trial designed to compare the effectiveness of concurrent versus delayed (by 6 months) treatment for smoking cessation among individuals receiving intensive alcohol dependence treatment (Joseph et al. 2003, 2004*a*). The smoking intervention included behavioral (i.e., a 1-hour, face-to-face intervention visit plus up to three follow- up visits) and pharmacologic (i.e., nicotine replacement therapy) components. Results indicated that although there were no differences in long-term smoking cessation rates between groups (approximately 16 percent of both groups were abstinent at 18 months), differences in alcohol use patterns at follow-up favored delayed treatment (Joseph et al. 2004*b*). Study participants who received concurrent smoking treatment had significantly lower alcohol abstinence rates at 6-, 12-, and 18-month follow-up visits (Joseph et al. 2004*b*). The time to first use of alcohol was shorter for those in the concurrent versus the delayed group, although the time to relapse was similar and there were no significant differences in the number of drinking days in the past 6 months (Joseph et al. 2004*b*).

The other randomized trial of smoking treatment timing followed 36 male veterans in a residential alcohol treatment program (Kalman et al. 2001). Smoking cessation treatment consisting of three 45-minute counseling sessions was administered either 2 or 6 weeks after admission. At the 20-week follow-up, 19 percent of the concurrent treatment group and 8 percent of the delayed smoking intervention group (difference not significant) reported smoking abstinence for the prior 7 days. However, other outcome data suggested greater rates of relapse to alcohol in the delayed smoking cessation intervention group (Kalman et al. 2001).

A potential explanation for the discrepancy in findings regarding effects on alcohol treatment outcomes is the variable timing for delivery of smoking intervention, even among the protocols described as concurrent treatment. For example, some researchers (Joseph et al. 1993, 2004*b*; Grant et al. 2003) provided smoking intervention early on in substance abuse treatment (within 1 to 2 weeks of treatment entry). In contrast, other studies provided smoking intervention after a brief period of sobriety or had an interval between the onsets of the two treatments. For example, Bobo and colleagues (1996) provided one brief counseling session at least 3 weeks after treatment admission. In another study (Bobo et al. 1998), only one of four smoking cessation counseling sessions took place before discharge from alcohol treatment; the majority were conducted postdischarge (i.e., at 8, 12, and 16 weeks). There may be important differences, in terms of outcomes, between initiating smoking treatment *immediately* and waiting several weeks. Although Kalman and colleagues (2001) and Hurt and colleagues (1994) provided concurrent treatment early on in alcohol treatment and did not observe a detriment to sobriety, these studies also had methodological limitations.^4^ Greater detriment to alcohol use outcomes among those receiving actual simultaneous smoking and alcohol treatment is consistent with review findings showing increased smoking abstinence when tobacco treatment is delivered following a longer period of sobriety (Prochaska et al. 2004; Sussman 2002).

## Summary and Suggestions for Future Research

Research to date suggests that alcohol- and nicotine-dependent smokers are interested in smoking cessation but prefer to address alcohol dependence prior to embarking on quitting smoking (e.g., Ellingstad et al. 1999). Overall, many alcohol-dependent smokers seem more interested in smoking treatment after a delay or period of sobriety, which has yet to be adequately quantified by research. Evidence suggests that effective smoking cessation interventions can be delivered but that their success often is short-term and dependent on treatment format (Prochaska et al. 2004). Experimental evidence also suggests potential for detriment to long-term sobriety among alcohol-dependent smokers who receive simultaneous smoking cessation intervention and alcohol treatment (Joseph et al. 2004*b*). Although this conclusion is supported by a large randomized controlled trial of smoking treatment timing, these results (Joseph et al. 2004*b*) were not consistent with a recent meta-analysis (e.g., Prochaska et al. 2004).

Potential reasons for the discrepancies in the existing literature include heterogeneous study designs, including nonrandomized studies. Other limitations include small sample sizes and, in some cases, predominantly male study populations (e.g., Cornelius et al. 1999; Grant et al. 2003; Kalman et al. 2001; Prochaska et al. 2004; Sussman 2002). The variability in the study populations and settings (e.g., in recovery, outpatient, residential, alcohol dependent or other substance dependent), methods of recruiting smokers (e.g., voluntary or mandatory), lack of consistent definitions (e.g., for “in recovery,” concurrent and sequential treatment), participant interest in smoking cessation, and smoking intervention formats (e.g., ranging from a brief counseling session to 16 weeks of treatment) add to the difficulty of comparing studies.

Many patients in substance use treatment or recovery do not receive concurrent or delayed nicotine dependence treatment, which is a pressing issue. This should be addressed by incorporating nicotine dependence treatment into aftercare for patients who are completing substance use treatment. With this program design, all smokers leaving treatment would be urged to participate in combined behavioral and pharmacologic treatment to quit smoking. Sequential treatment delivery has practical limitations, however. For example, if smoking cessation is not offered concurrently with alcohol treatment, it may be difficult to interest individuals in treatment even 4 to 6 weeks later and maintain their participation, as several studies have found (Grant et al. 2003; Kalman et al. 2001; Joseph et al. 2004*b*). Based on the literature to date, it is difficult to know conclusively that concurrent treatment should be avoided, but this is a possibility. Concurrent tobacco dependence treatment should not be denied, however, if requested by patients.

Although the health threats of smoking are clear, many unanswered questions remain about how to best approach smoking cessation in alcohol- and substance-dependent populations. In particular, research based on statistically strong randomized controlled trials of fully integrated alcohol and tobacco treatment and conducted in diverse samples is needed. We need additional data on the most effective smoking treatment modalities for this population and on techniques for orchestrating the delivery of sequential smoking cessation treatment. These may include investigations of traditional and alternative treatment formats (including telephone care, integration with 12- step programs, and referral to primary care providers). Further detailed study of the effects of the timing of treatment is also needed. For example, research is needed to compare smoking cessation interventions delivered immediately after substance use treatment with interventions delivered 2, 4, and 8 weeks later, as well as with interventions delivered 6 months later. Finally, it is essential to gather additional information about the role that individual difference variables such as degree of alcohol or nicotine dependence, coping skills, mental health, and gender may play in alcohol and tobacco cessation.

## Footnotes

1 Researchers use randomization, or random assignment of study participants to intervention and control groups, to eliminate bias.

2 Among cocaine users, those in the intervention group had significantly worse outcomes when people who could not be followed up because they did not respond to researcher requests for information were categorized as treatment failures (i.e., nonrespondents).

3 The meta-analysis (Prochaska et al. 2004) included 12 treatment studies, 5 of which focused on patients in alcohol treatment, including Bobo et al. 1995, 1998; Cornelius et al. 1997, 1999; Grant et al. 2003; and Kalman et al. 2001.

4 One study (Kalman et al. 2001) had limited power to detect significant differences because of a small sample size, and one (Hurt et al. 1994) used a nonrandomized design.
